# Editorial: Connecting Music and Body Movement: Choreographic Approach of Performance

**DOI:** 10.3389/fpsyg.2022.875214

**Published:** 2022-05-16

**Authors:** Zélia Chueke

**Affiliations:** ^1^Department of Music and Visual Arts, Universidade Federal do Paraná (UFPR), Curitiba, Brazil; ^2^Institut de recherche en Musicologie (IReMus) UMR8223/CNRS, BnF, Sorbonne Université, MCC, Paris, France

**Keywords:** performance analysis, sound quality, sound identity, choreography, ergonomics

## Introduction

In the field of musical performance, going beyond the basic playing mechanism, in which training is contemplated by different approaches of technique, this Research Topic aims to explore what we chose to call “choreography,” consisting of movements that make it possible for musicians to produce different qualities of sound in the same passage. Individual sound quality is, therefore, envisaged and cultivated, defining the performer's identity, independently from the pitch, duration, articulation, dynamics, and other aspects belonging to the area of interpretation. Additionally, in finding the most comfortable way of producing the imagined—and personal—sound, ergonomic aspects are immediately associated with the concept of choreography proposed. Aiming to enlarge perspective terrain for music performance analysis, different subjects were suggested when calling for submissions, trying to access objectiveness and/or intuition regarding decisions about sound quality, the tripartite relationship “Choreography-Ergonomics-Sound Production,” body awareness during practice and performance as well as technological resources making this process visible and observable. Presenting various and enriching perspectives to access the proposed line of research, the overview, hereby presented, reinforces the invaluable contribution provided to this area of study—extended to dance in one of the articles—by research works conducted from the performer's standpoint.

“*Mirror Neuron Activity During Audiovisual Appreciation of Opera Performance*” by Tanaka explored neurophysiological mechanisms implied in perception-action combination during an opera performance where the public was formed by singers. In this study, the engagement of the mirror-neuron system by the performance's perception was measured by EGG (electroencephalography), demonstrating how close singers' performance on stage was to what was actually experienced by those attending the concert.

From another standpoint, Dalmazzo et al. classified arm gestures exploring violinists' daily practice in “*Applying Deep Learning Techniques to Estimate Patterns of Musical Gesture*.” Their research was made possible by what they chose to call “different architectures,” applying CNN (*convolutional neural networks*) models in the initial analysis.

Regarding the relationship between the quality of sound and the pianist's identity, in “*Teaching and Learning of Piano Timbre Through Teacher-Student Interactions in Lessons*,” Li and Timmers demonstrated that building and cultivating this particular and essential element of tone production can be guided and encouraged by teachers by means of achieving conscient mind-body integration. Rigorous methodology and references signed by specialists such as Neuhaus (1993), Hallam (1998), and Rosen (2002), among others illustrates the variety of domains implied in the activity being investigated.

The relevance of the exact timing regarding movement in space for both musical performance and dance is explored by Ladda et al. in “*Multimodal Sensory-Spatial Integration and Retrieval of Trained Motor Patterns for Body Coordination in Musicians and Dancers*.” Focusing on the storage of movement patterns by the lateral prefrontal cortex (lPFC) and the generation of movements by the intraparietal sulcus (IPS) the authors investigate the modification of these trajectories in order to optimize performances of dance and music. A rich list of references supported the output of this Hypothesis and Theory article. The ideas of Keller et al. (2014) support their initiative considering temporally precise rhythmic interpersonal coordination which, according to this author, “requires three core cognitive-motor skills: anticipation, attention, and adaptation.” Refining sensorimotor behavior and precision of timing in the visual and auditory domain are obviously implied, based on the study by Cicchini et al. (2012) directly related to the proposed model “motor learning through motor imaging.”

Aiming to access music comprehension during performance, that is to say, music that emanates from performance, Visi et al. proposed a kind of multimodal analysis, as holistic as musical performance itself, combining qualitative and quantitative kinds of approach. In their article “*Method Development for Multimodal Data Corpus Analysis of Expressive Instrumental Music Performance*,” they consider the establishment of a specific data compilation method in order to access instrumental performance from numerous perspectives. Concomitantly, they tried to develop musical composition techniques taking profit from cross-modal relations as well as elements that emerge from the analysis of a multilevel and multimodal corpus from a larger perspective that combines arts and human science.

Finally, the article “*Connecting Free Improvisation Performance and Drumming Gestures Through Digital Wearables*” signed by Pras et al. presents a collaboration project, where designers, a music producer conducting research on improvisation, and professional musicians intended to create a modular concept of wearable interfaces that control musical parameters in drumming with electronics. Three vest prototypes illustrate their initiative, corroborating the importance of gestures in the making of improvised performances underlined by Mazzola and Cherlin (2008).

Besides promoting precious input from the perspectives of different areas of expertise, articles introduced by this Research Topic indicated the relevance of technology resources regarding the scientific approach and objectiveness of decisions concerning performance choreography from the starting point of sound imagination. It is then possible to envisage accessing the most detailed anatomic aspects of musical performance, instigating new research initiatives not forgetting the non-explainable aspects inherent to the art universe.

## Author Contributions

The author confirms being the sole contributor of this work and has approved it for publication.

## Conflict of Interest

The author declares that the research was conducted in the absence of any commercial or financial relationships that could be construed as a potential conflict of interest.

## Publisher's Note

All claims expressed in this article are solely those of the authors and do not necessarily represent those of their affiliated organizations, or those of the publisher, the editors and the reviewers. Any product that may be evaluated in this article, or claim that may be made by its manufacturer, is not guaranteed or endorsed by the publisher.
